# Immune mechanism of low bone mineral density caused by ankylosing spondylitis based on bioinformatics and machine learning

**DOI:** 10.3389/fgene.2022.1054035

**Published:** 2022-11-18

**Authors:** Ding Zhang, Jia Liu, Bing Gao, Yuan Zong, Xiaoqing Guan, Fengyi Zhang, Zhubin Shen, Shijie Lv, Li Guo, Fei Yin

**Affiliations:** ^1^ Department of Spine Surgery, China-Japan Union Hospital of Jilin University, Changchun, Jilin, China; ^2^ Department of Orthodontics, Hospital of Stomatology, Jilin University, Changchun, Jilin, China; ^3^ Department of Toxicology, School of Public Health, Jilin University, Changchun, Jilin, China

**Keywords:** ankylosing spondylitis, low bone mineral density, bioinformatics, machine learning, immune infiltration, community discovery

## Abstract

**Background and Objective:** This study aims to find the key immune genes and mechanisms of low bone mineral density (LBMD) in ankylosing spondylitis (AS) patients.

**Methods:** AS and LBMD datasets were downloaded from the GEO database, and differential expression gene analysis was performed to obtain DEGs. Immune-related genes (IRGs) were obtained from ImmPort. Overlapping DEGs and IRGs got I-DEGs. Pearson coefficients were used to calculate DEGs and IRGs correlations in the AS and LBMD datasets. Louvain community discovery was used to cluster the co-expression network to get gene modules. The module most related to the immune module was defined as the key module. Metascape was used for enrichment analysis of key modules. Further, I-DEGs with the same trend in AS and LBMD were considered key I-DEGs. Multiple machine learning methods were used to construct diagnostic models based on key I-DEGs. IID database was used to find the context of I-DEGs, especially in the skeletal system. Gene–biological process and gene-pathway networks were constructed based on key I-DEGs. In addition, immune infiltration was analyzed on the AS dataset using the CIBERSORT algorithm.

**Results:** A total of 19 genes were identified I-DEGs, of which IFNAR1, PIK3CG, PTGER2, TNF, and CCL3 were considered the key I-DEGs. These key I-DEGs had a good relationship with the hub genes of key modules. Multiple machine learning showed that key I-DEGs, as a signature, had an excellent diagnostic performance in both AS and LBMD, and the SVM model had the highest AUC value. Key I-DEGs were closely linked through bridge genes, especially in the skeletal system. Pathway analysis showed that PIK3CG, IFNAR1, CCL3, and TNF participated in NETs formation through pathways such as the MAPK signaling pathway. Immune infiltration analysis showed neutrophils had the most significant differences between case and control groups and a good correlation with key I-DEG.

**Conclusion:** The key I-DEGs, TNF, CCL3, PIK3CG, PTGER2, and IFNAR1, can be utilized as biomarkers to determine the risk of LBMD in AS patients. They may affect neutrophil infiltration and NETs formation to influence the bone remodeling process in AS.

## Introduction

Ankylosing spondylitis (AS) is a inflammatory rheumatic disease, mainly affecting the axial skeleton, causing characteristic inflammatory back pain, leading to structural and functional disorders and declining quality of life (Braun and Sieper, 2007). The enthesitis of AS increases new bone formation and spinal ankylosis. Meanwhile, the loss of bone trabecula in the vertebral body of AS patients lowered local bone mineral density and caused osteoporosis (Klingberg et al., 2012; Nam et al., 2021). Interestingly, the opposite trend of bone metabolism reduced the spinal ability to resist impact and strain, increasing the risk of spinal fracture, then increasing the probability of spinal cord and nerve root injury and mortality in patients with AS.

As an essential risk factor for vertebral fracture and a common complication of AS, low bone mineral density (LBMD) has attracted the attention of many scholars. Generally speaking, LBMD or osteoporosis occurs mainly in postmenopausal women. In contrast, secondary osteoporosis induced by AS primarily occurs in young and middle-aged men (Sieper and Poddubnyy, 2017), indicating that, in addition to age and hormone levels, reduced BMD in AS has its distinct mechanism (Bessant and Keat, 2002). The LBMD of the femoral neck and lumbar vertebrae in patients with AS was associated with disease activity (Magrey and Khan, 2010; Kang et al., 2018). Previous studies have found that LBMD in AS was related to local inflammation (Liu et al., 2022). Disorders of redox biomarkers, such as increased advanced oxidation protein products and decreased glutathione peroxidase, also affect the level of LBMD (Wang et al., 2015).

Recent studies on the mechanism of LBMD caused by AS have focused on immunity and inflammation. There was a significant correlation between LBMD and bone turnover markers, osteoprotegerin, proinflammatory cytokines, and acute phase reactants such as CRP and ESR in AS patients, suggesting that inflammatory mediators may be involved in the pathogenesis of LBMD in AS (Kim et al., 2006; Ranganathan et al., 2017; Wu et al., 2021; Rademacher et al., 2022). It has been demonstrated that cytokines, such as IL-6 and TNF, play a vital role in AS inflammation and may be involved in bone destruction in AS (Lee et al., 2022; Rademacher et al., 2022). In addition, IL-17, a T-cell cytokine that promotes osteoclast production and bone resorption, is proposed as one of the mechanisms of increased bone destruction in AS (Tsukazaki and Kaito, 2020). Unfortunately, the mechanism by which AS induces LBMD is not fully understood. Therefore, exploring the role of immune-related genes in AS-induced LBMD is helpful in accurately judging the fracture risk of AS patients, guiding drug development and clinical management.

**FIGURE 1:**
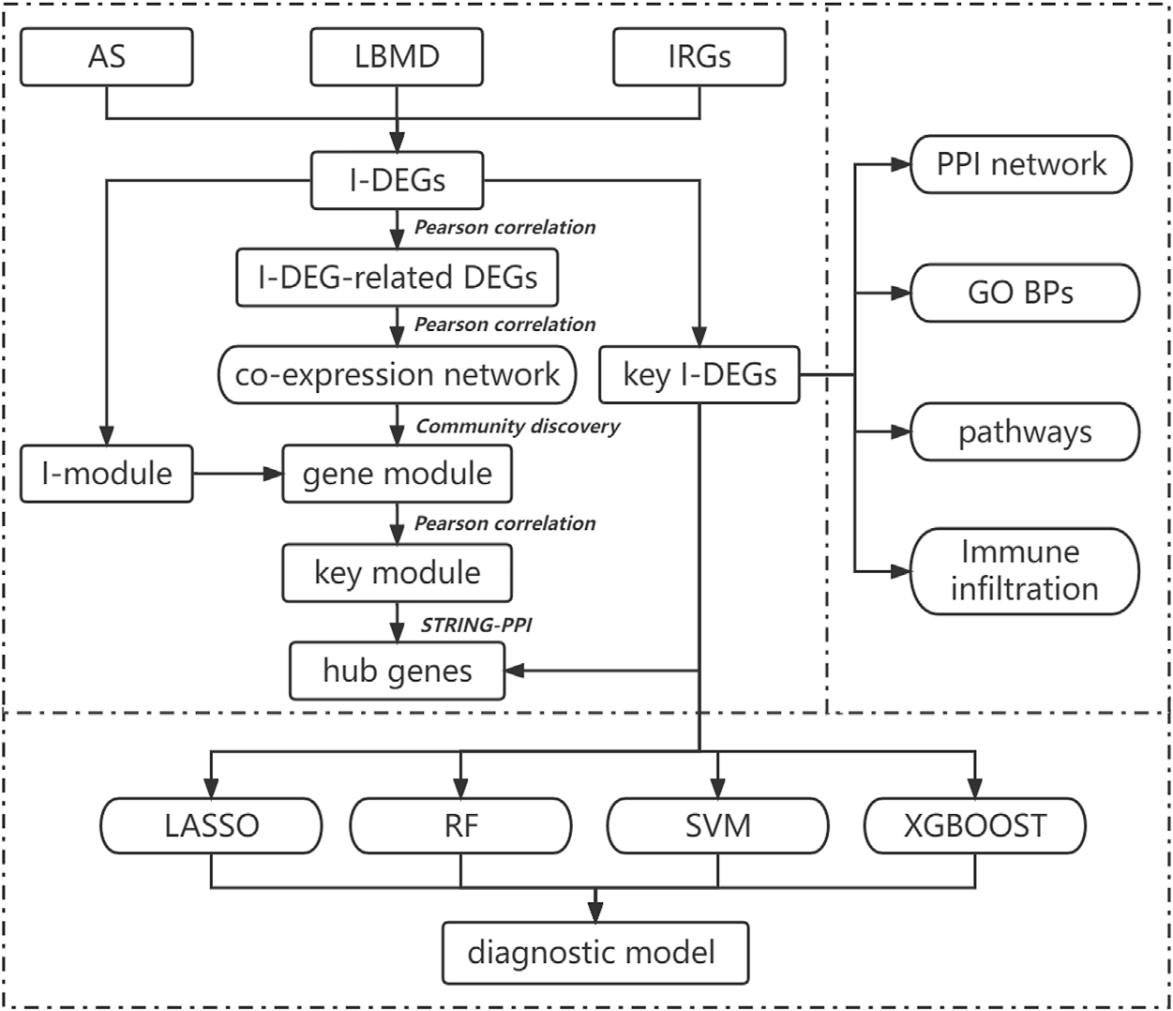
Flow-chart of datasets analysis in this paper.

The purpose of our study is to comprehensively use bioinformatics methods to explore the specific mechanism of immune-related genes in AS-induced LBMD and its relationship with immune cells. At the same time, machine learning methods were used to establish diagnostic models to evaluate the risk of LBMD in AS patients and guide clinical treatment. The flow chart of our study is shown in Figure 1.

## Materials and methods

### Data download and preprocessing

We obtained the AS datasets (GSE25101, GSE73754) and the LBMD datasets (GSE56815, GSE2208) from the GEO database (Table 1). All analyses were performed using R software (version 4.0.2).

**TABLE 1:** The information of datasets.

Datasets	Disease	Platform	Control	Case
GSE25101	AS	GPL6947	16	16
GSE73754	AS	GPL10558	20	52
GSE56815	LBMD	GPL96	40	40
GSE2208	LBMD	GPL96	10	9

Overlapping genes between each disease dataset were identified. For AS, the intersection of the genes analyzed in GSE25101 and GSE73754 was determined, and the same for LBMD (GSE56815, GSE2208). The expression of these overlapping genes was extracted from each dataset. We used the ComBat method to eliminate batch bias using the sva package (version 3.36.0).

### Identification of DEGs and I-DEGs

The corrected AS and LBMD datasets were subjected to differential expression analysis (DEA) by the limma package (version 3.44.3). We defined the genes with *p*-values < 0.05 as differential expressed genes (DEGs). The ggplot2 package (version 3.3.2) and pheatmap package (version 1.0.12) were used to depict gene expression by heatmaps and volcano plots.

The immune-related genes (IRGs) were obtained from the ImmPort database (Bhattacharya et al., 2018). The AS DEGs, LBMD DEGs, and IRGs were taken intersection to obtain the dysregulated IRGs in both AS and LBMD, and we called them immune-DEGs (I-DEGs).

### Correlation analysis and co-expression network construction

The correlation between I-DEGs and DEGs in AS and LBMD was analyzed by calculating the Pearson coefficient in the Hmisc package (version 4.4.1). The DEGs related to I-DEGs were obtained with moderate correlation (*p*-values < 0.05 and | r | > 0.40). In addition, we constructed a co-expression network of I-DEG-related DEGs with strong correlation (*p*-values < 0.05 and | r | > 0.70).

### Community discovery analysis

Louvain is a clustering algorithm frequently employed in network clustering, notably in human PPI networks, intending to decrease external connections while encouraging intra-community connections (Smith et al., 2020). We clustered the co-expression network using the Louvain algorithm in the igraph package (version 1.2.5), where the weight was set to | r |. The modules with less than 30 genes were deleted, and the Cytoscape software (version 3.9.0) was used for visualization.

### Correlation analysis of modules

Module eigengenes (MEs) were defined as the genes in the first principal component of gene modules using the PCA algorithm. The I-DEGs were defined as the immune module as a whole. The correlation between the MEs of I-DEG-related DEGs modules and the ME of the immune module was analyzed by the Pearson correlation coefficient and visualized by the corrplot package (version 0.84). Among them, the modules with the highest correlation were considered the key modules and studied in subsequent analyses.

### Functional enrichment analysis

The Metascape database (Zhou et al., 2019) was used to perform GO and KEGG functional enrichment analysis for the key module genes. The criteria of the analyses were *p*-values < 0.01, min (overlap) = 3, and min (enrichment) = 1.5, and the outcomes were visualized using heatmaps.

### PPI network construction and hub genes identification

The PPI network of the key modules was constructed by the STRING database (Szklarczyk et al., 2021) and visualized by Cytoscape. The cytohubba plug-in was used to find the top10 hub genes of the key modules.

The I-DEGs with the same expression trend in AS and LBMD were considered the key I-DEGs. They were imported into the STRING database with the hub genes and visualized by Cytoscape to explore the relationship between the key I-DEGs and hub genes.

### Construction of diagnostic model by machine learning

To explore the function of key I-DEGs as a whole in more depth, we designated them as the key signature. The AS and LBMD datasets were split into training and testing sets with a 7:3 ratio. The Glmnet package (version 4.0.2), randomForest package (version 4.6.14), XGBoost package (version 1.4.1.1) and e1071 package (version 1.7.3) were used to construct the diagnostic model with the key signature. We get the LASSO model and the corresponding feature coefficients by performing the least absolute shrinkage and selection operator (LASSO) binomial analysis in the Glmnet package. The penalty parameter (*λ*) was decided by the minimum criteria. The randomForest package was used to build a random forest model for the key signature, and the decreasing accuracy method (Gini coefficient method) was used to obtain the feature importance values. The XGBoost package was used to build the XGBoost model. The XGBoost model was a tree-based algorithm that can provide each feature importance score and rank them. The e1071 package was used to build the support vector machine (SVM) model, a typical supervised machine learning method.

### In-depth study of key I-DEGs

To explore the interaction context of key I-DEGs, especially in the skeletal system, we imported key I-DEGs into the IID database and constructed the PPI network in all tissue and bone. We called the genes that connected key I-DEGs bridge genes, which may help us understand the context of key I-DEGs and make the gene interaction connection abundant.

To investigate the pathogenic mechanism and better comprehend the function of key I-DEGs, the key I-DEGs were imported into the GO (Carbon et al., 2009) and KEGG database (Kanehisa and Goto, 2000) to obtain human biological processes and pathways comprising at least two genes. To provide a better explanation for the findings, the biological processes acquired were combined and clustered by ClueGo (Bindea et al., 2009). KEGG database was utilized to explore the common pathways map, getting potential key I-DEG and pathway associations.

### Immune infiltration

The CIBERSORT (Newman et al., 2015) algorithm was used to acquire the immune infiltration matrix in the AS datasets. Using the ggplot2 package, each sample and group’s immune infiltration was shown graphically. The differences between the two groups were examined by the Wilcoxon test. Using the corrplot package, we plotted correlation heatmaps to demonstrate the association between the 22 immune cells and between key I-DEGs and immune cells.

## Result

### Identification of DEGs and I-DEGs

**FIGURE 2:**
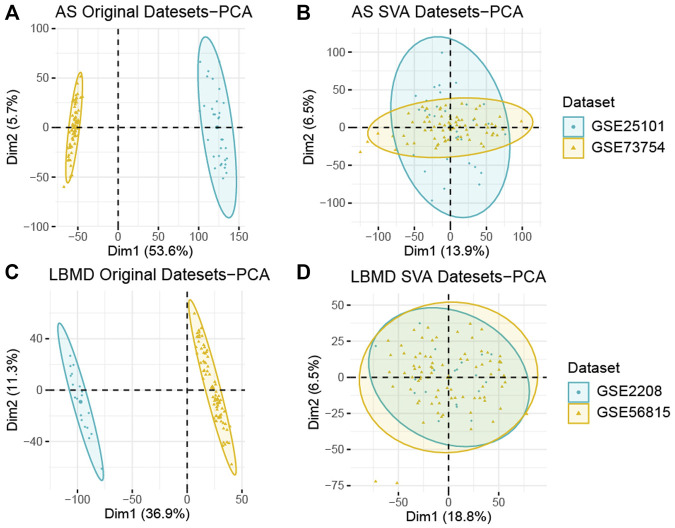
**(A)** PCA analysis results of AS before batch correction; **(B)** PCA analysis results of AS after batch correction; **(C)** PCA analysis results of LBMD before batch correction; **(D)** PCA analysis results of LBMD after batch correction.

**FIGURE 3:**
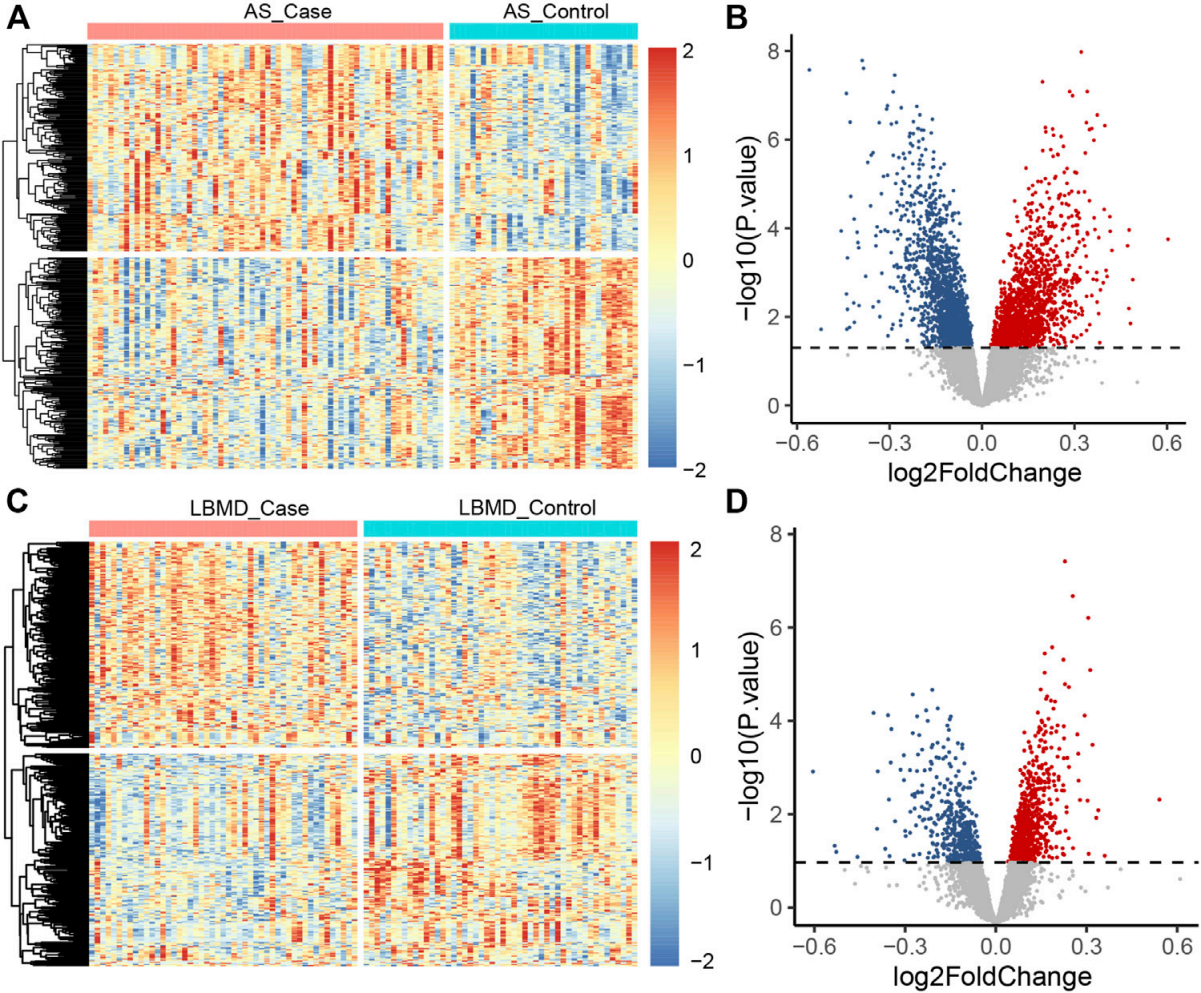
**(A)** The heatmap plot of AS DEGs; **(B)** The volcano plot of AS DEGs; **(C)** The heatmap plot of LBMD DEGs; **(D)** The volcano plot of LBMD DEGs.

**FIGURE 4:**
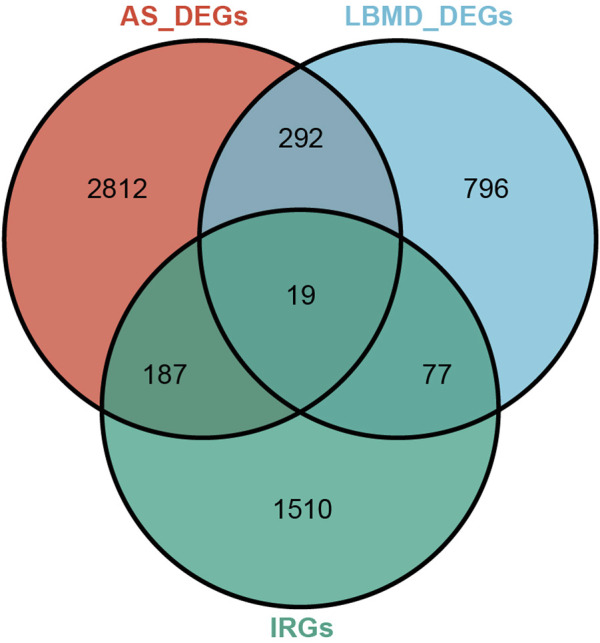
Venn diagram of DEGs and IRG.

After batch correction, the differences between samples in AS and LBMD datasets were significantly reduced (Figure 2). Based on DEA, we got DEGs of AS and LBMD, including 1,492 up-regulated and 1818 down-regulated genes in AS, and 730 up-regulated and 454 down-regulated genes in LBMD. Figure 3 showed the expression of DEGs. From the ImmPort database, we obtained 1793 IRGs (Supplementary Table S1). Figure 4 showed that a total of 19 I-DEGs were identified. They were not only common DEGs of AS and LBMD, but also IRGs. These I-DEGs were used for our subsequent analysis in depth.

### Co-expression network construction and analysis of modules discovery and correlation

**FIGURE 5:**
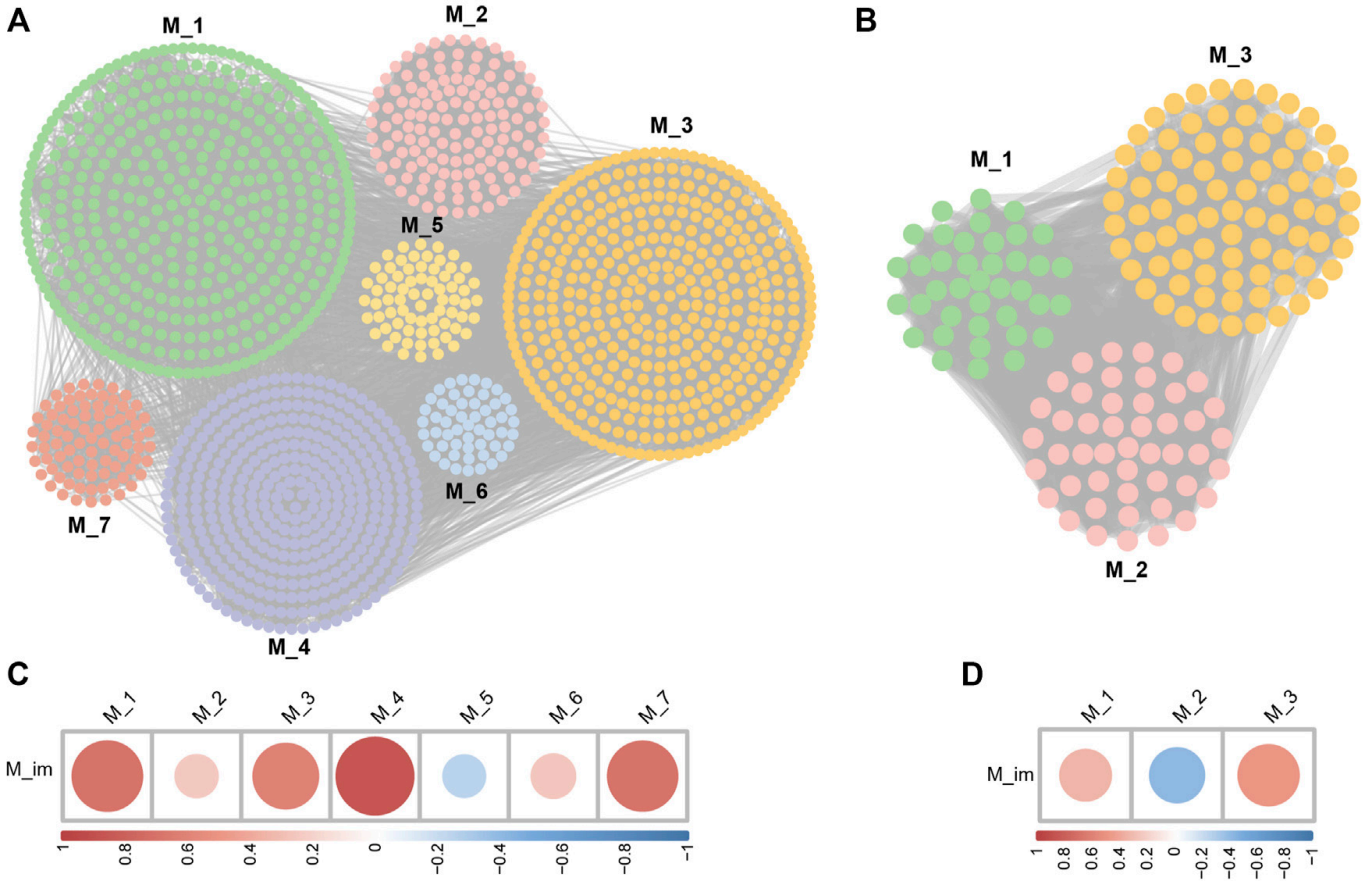
**(A)** The community discovery clustering network of AS; **(B)** The community discovery clustering network of LBMD; **(C)** Correlation between the immune module and AS modules; **(D)** Correlation between the immune module and LBMD modules.

Through the Pearson correlation analysis, we obtained 2,227 DEGs related to I-DEGs in AS and 735 in LBMD (Supplementary Table S2). Furthermore, the co-expression networks of these genes were constructed in AS and LBMD, respectively. By the Louvain community discovery algorithm, the gene modules with less than 30 genes were deleted, and the gene co-expression network of AS was re-clustered into seven modules. Similarly, LBMD was re-clustered into three modules (Figures 5A,B). Density is an assessment metric for measuring the connectivity degrees of the network. The density of these modules was shown in Table 2. It was noted that the density of these modules was higher than that of the co-expression network (AS: 0.01673044, LBMD: 0.05024927). It demonstrated that the multilevel algorithm’s partitioning result was reliable. Figures 5C,D showed that M4 was the most related to the immune module in AS and M3 in LBMD. These modules were called key modules and were closely related to immunity which may play an essential role in AS and LBMD.

**TABLE 2:** The density of each module.

Disease	Module	Graph density
AS	1	0.034032719
	2	0.510005528
	3	0.074860982
	4	0.06239988
	5	0.23943662
	6	0.301886792
	7	0.116526611
LBMD	1	0.417004049
	2	0.299722479
	3	0.077452668

### Functional analysis of key modules

**FIGURE 6:**
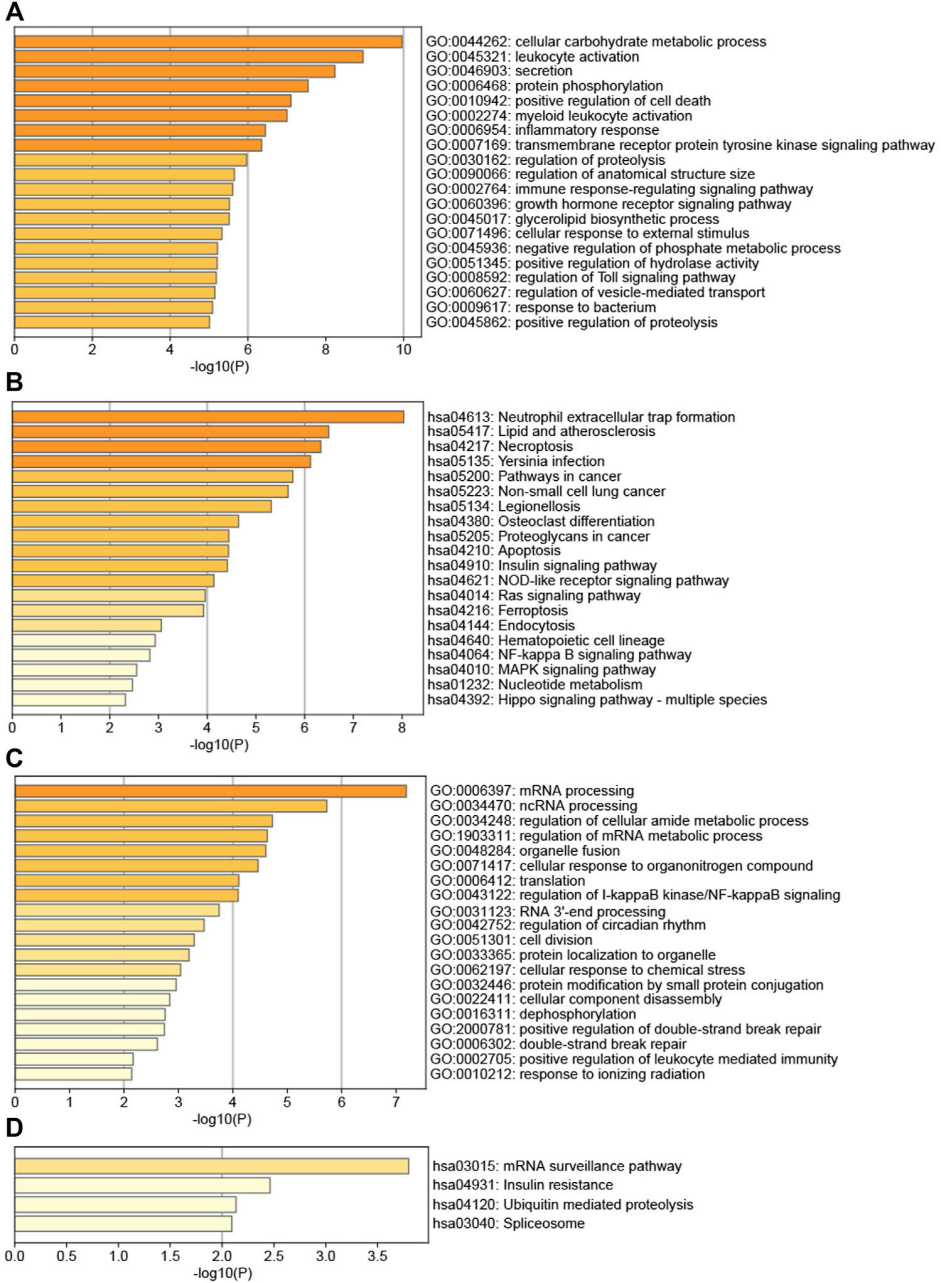
**(A)** TOP20 GO BP terms of AS key module; **(B)** TOP20 KEGG pathways of AS key module; **(C)** TOP20 GO BP terms of LBMD key module; **(D)** TOP4 KEGG pathways of LBMD key module.

Figure 6A showed that the genes of AS key module AS-M4 were mainly involved in several GO BPs, for example, cellular carbohydrate metabolic process, leukocyte activation, secretion, protein phosphorylation, positive regulation of cell death, myeloid leukocyte activation, and inflammatory response. Meanwhile, AS-M4 genes were mainly enriched in several pathways (Figure 6B), Neutrophil extracellular trap formation, Lipid and atherosclerosis, Necroptosis, *Yersinia* infection, Pathways in cancer, Non-small cell lung cancer, Legionellosis, Osteoclast differentiation, Proteoglycans in cancer, Apoptosis. This module was closely related to immunity, such as immune cell activation and inflammatory response. Interestingly, osteoclast differentiation was one of its significant pathways, confirming its vital role in affecting bone homeostasis.

Figure 6C showed that the biological processes of LBMD key module LBMD-M3 were mainly enriched in mRNA processing, ncRNA processing, regulation of cellular amide metabolic process, regulation of mRNA metabolic process, organelle fusion, cellular response to organonitrogen compound, translation, regulation of I-kappaB kinase/NF-kappaB signaling, RNA 3′-end processing, regulation of circadian rhythm, cell division, protein localization to organelle, cellular response to chemical stress, etc. Meanwhile, LBMD-M3 genes were mainly enriched in several pathways (Figure 6D), mRNA surveillance pathway, Insulin resistance, Ubiquitin mediated proteolysis and Spliceosome. Interestingly, we found this module participated in the regulation of I-kappaB kinase/NF-kappaB signaling, while the receptor activator of NF-kappaB (RANK) had been fully recognized as causing the osteoclast precursor to differentiate into a preosteoclast, which was an essential mechanism of bone destruction (Weitzmann, 2017).

### Identification of hub genes and key I-DEGs

The PPI network of AS-M4 was obtained from the STRING database, including 289 nodes and 787 edges, and the PPI network of LBMD-M3, including 56 nodes and 89 edges (Supplementary Figures S1A,C). The top10 hub genes were identified by the cytohubba plug-in (Supplementary Figures S1B,D).

We further explored the expression of I-DEGs and found that five genes (TNF, CCL3, IFNAR1, PIK3CG, and PTGER2) showed the same trend in AS and LBMD. By examining the expression of IRGs in AS and LBMD, we found that CCL3, PTGER2, and TNF showed a common down-regulation trend in both diseases, while IFNAR1 and PIK3CG showed a common up-regulation trend. These five genes were considered key I-DEGs.

**FIGURE 7:**
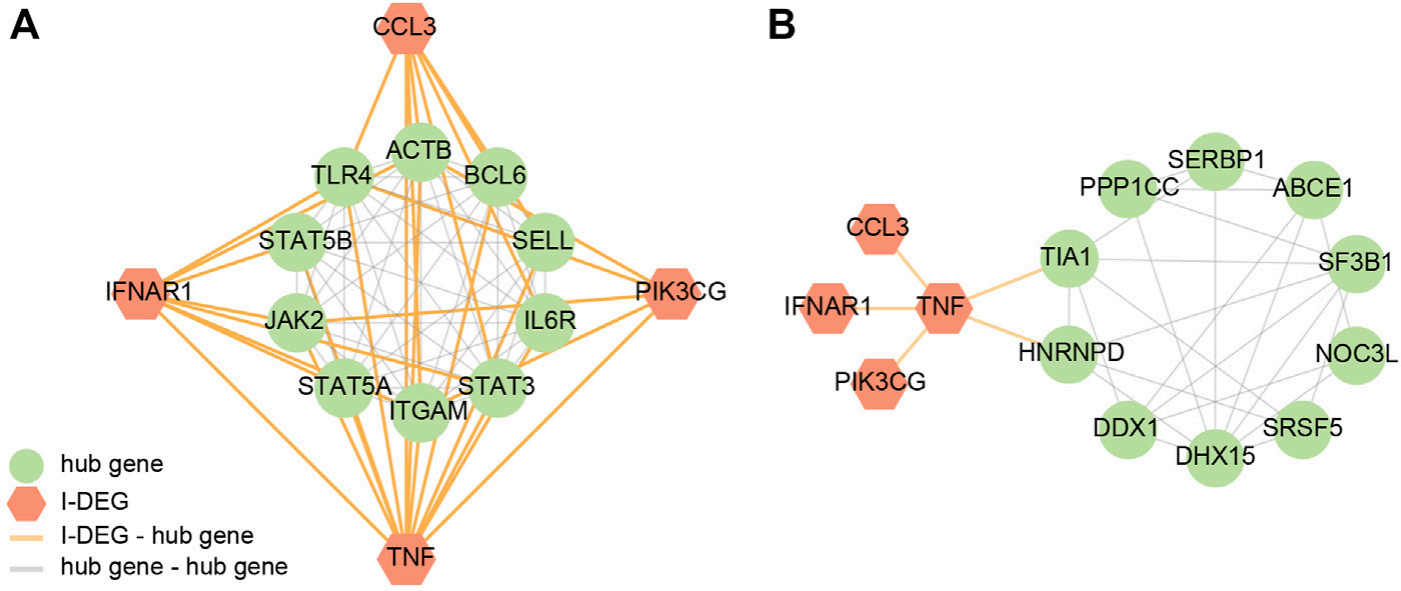
**(A)** The PPI network of key I-DEGs and AS key module’s hub genes; **(B)** The PPI network of key I-DEGs and LBMD key module’s hub genes.

By building the PPI networks of key I-DEGs and hub genes, respectively (Figure 7), we found that key I-DEGs had a good relationship with AS-M4 hub genes, while TNF can be related to UBE2N and TIA1 in LBMD.

### Diagnostic model construction

**FIGURE 8:**
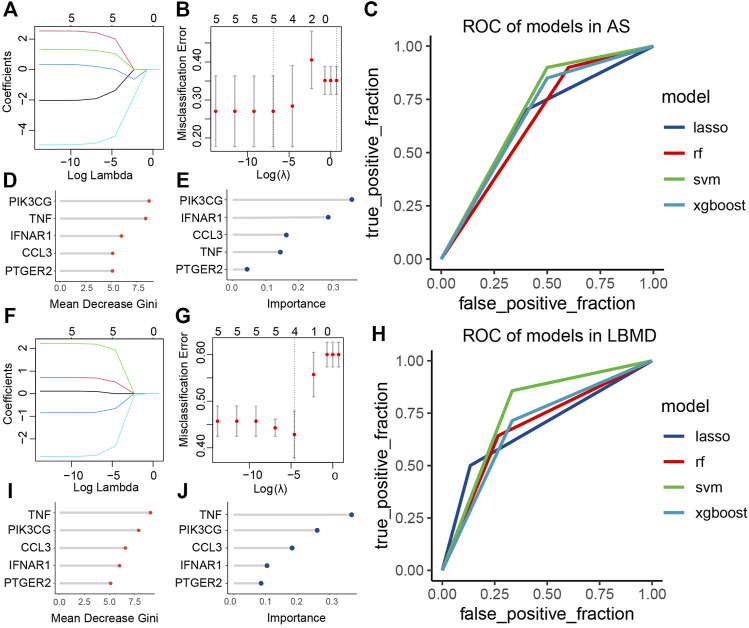
**(A)** LASSO regression of five genes in AS; **(B)** Cross-validation for tuning the parameter selection in the LASSO regression of AS; **(C)** ROC curve analysis of models in AS; **(D)** The importance of each feature in RF of AS; **(E)** The importance of each feature in XGBoost of AS; **(F)** LASSO regression of four genes in LBMD; **(G)** Cross-validation for tuning the parameter selection in the LASSO regression of LBMD; **(H)** ROC curve analysis of models in LBMD; **(I)** The importance of each feature in RF of LBMD; **(J)** The importance of each feature in XGBoost of LBMD.

In AS, the LASSO model composed of five features was constructed according to the optimal λ value (LASSO score: 1.92exp (CCL3) + 2.42exp (IFNAR1) + 1.25exp (PIK3CG) + 0.2exp (PTGER2)-4.84exp (TNF) (Figures 8A,B). In LBMD, four features constituted the LASSO model (LASSO score: 0.52exp (IFNAR1) + 1.93exp (PIK3CG)-0.65exp (PTGER2)-2.31exp (TNF)) (Figures 8F,G).

We found that key I-DEGs as a whole had a good diagnostic value in LBMD, among which the SVM model had the highest AUC value of 0.76 (Figure 8H). Interestingly, the signature also had an excellent diagnostic value in AS (Figure 8C), further illustrating the critical relationship between AS and LBMD. The specific AUC values of the four models were shown in Table 3.

**TABLE 3:** AUC of each model in AS and LBMD.

Model	As	LBMD
LASSO	0.65	0.68
RF	0.65	0.69
SVM	0.7	0.76
XGBoost	0.675	0.69

In LBMD RF and XGBoost models, we found that both TNF and PIK3CG were the top2 most important feature (Figures 8D,E), and PIK3CG was the most important in AS RF and XGBOOST models, and TNF was the second in RF and the fourth in XGBoost (Figures 8I,J). It undoubtedly confirmed the role of TNF and PIK3CG in AS and LBMD, and was worthy of in-depth discussion.

### Connections between key I-DEGs

**FIGURE 9:**
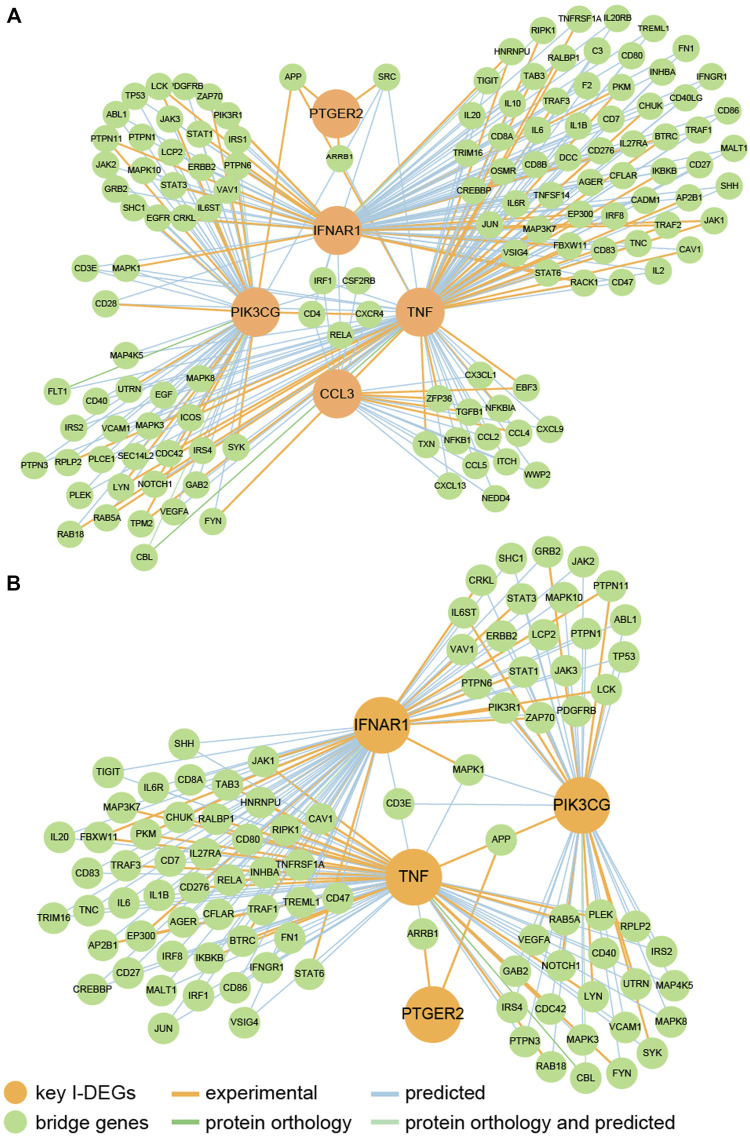
**(A)** The PPI network of key I-DEGs connected by bridge genes; **(B)** The PPI network of key I-DEGs connected by bridge genes in the skeletal system.

We identified that key I-DEGs potentially can interact with one another through bridge genes (Figure 9A). Furthermore, it was found that IFNAR1, PIK3CG, TNF, and PTGER2 can interact in the skeletal system (Figure 9B). This complements the interaction between key I-DEGs, especially in bone homeostasis.

**FIGURE 10:**
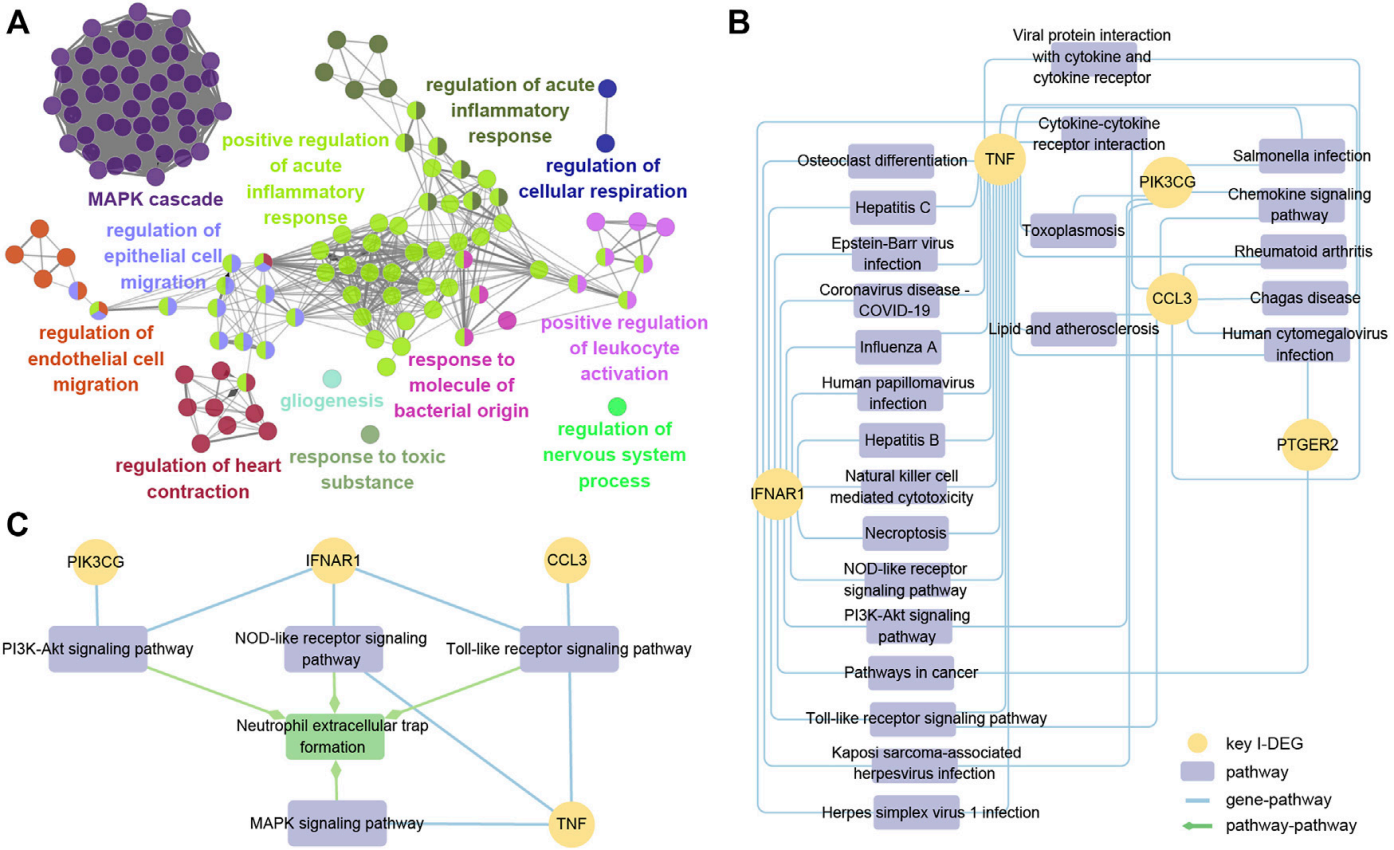
**(A)** ClueGO fusion and cluster result of biological processes involved in key I-DEGs; **(B)** Key I-DEGs-pathway networks; **(C)** The relationship between NETs formation pathway and key I-DEGs.

To get a deeper understanding of the function of key I-DEGs, we integrated them into the GO database and identified 305 biological processes, including at least two key I-DEGs (Supplementary Table S3). The results of ClueGO indicated that 305 biological processes were fused into 131 based on the relationship between GO terms, with MAPK cascade and positive control of acute inflammatory response comprising the majority (Figure 10A).

To further investigate the specific mechanism of key I-DEGs, the key I-DEGs were imported into the KEGG database, and we identified 24 pathways comprising at least two genes after filtering. (Figure 10B). Interestingly, TNF and IFNAR1 were involved in osteoclast differentiation, suggesting that key I-DEGs may cause bone mass loss in AS.

As the most significant pathway of the AS key module, neutrophil extracellular traps (NETs) formation attracted our attention. By viewing the map of NETs formation pathway in the KEGG database, we found that there were four pathways, including PI3K-Akt signaling pathway, NOD-like receptor signaling pathway, Toll-like receptor signaling pathway, and MAPK signaling pathway, participating in NETs formation, and also closely related to the four key I-DEGs (PIK3CG, IFNAR1, CCL3, and TNF) (Figure 10C). NETs formation was not only involved in the pathogenesis of AS, but may also be the key to these key I-DEGs connections.

### Immune infiltration

**FIGURE 11:**
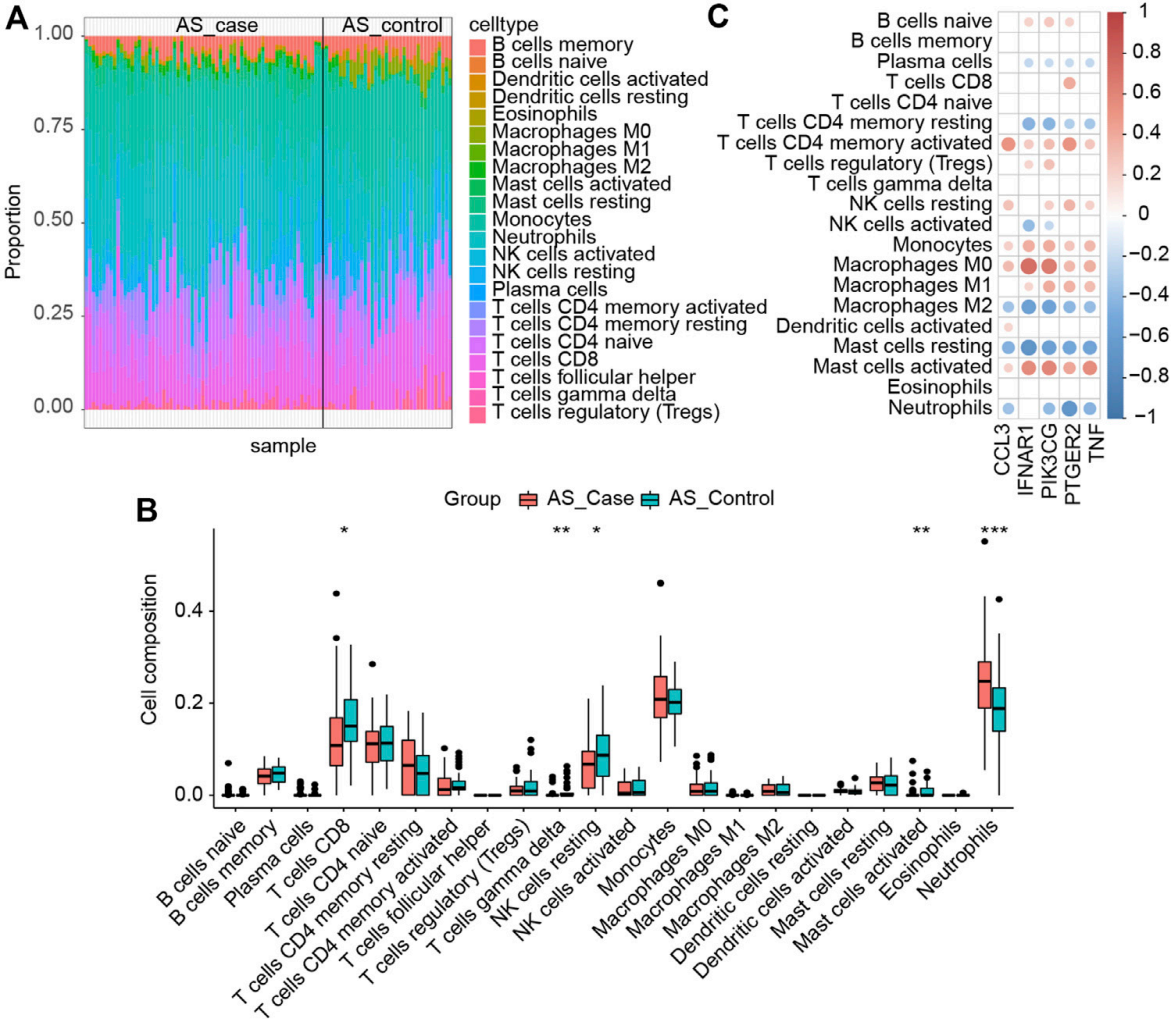
**(A)** Immune infiltration of each sample; **(B)** Comparison of immune cells between the two groups (∗: *p*-value < 0.05, ∗∗: *p*-value < 0.01, ∗∗∗: *p*-value < 0.001); **(C)** Correlation between immune cells and key I-DEGs.

We analyzed the immune infiltration of AS datasets using the CIBERSORT algorithm to further investigate immune cells’ function in AS and the impact of key I-DEGs on it. Figure 11A provided immune infiltration of the samples. Five immune cell subpopulations revealed significant differences between case and control groups (*p*-values < 0.05). Compared with the control group, the case group contained a higher proportion of neutrophils, lower T cells CD8, T cells gamma delta, NK cells activated and mast cells activated, and neutrophils were the most significant (*p*-value < 0.001) (Figure 11B). Figure 11C showed that all the key I-DEGs negatively correlated with macrophage M2, and IFNAR1, PIK3CG, PTGER2 and TNF were positively correlated with macrophage M1. It was a rather interesting outcome indicating key I-DEGs can cause an imbalance of macrophages M1/M2 cells in AS, which has been shown to be associated with bone loss (Wang et al., 2022). It suggests that key I-DEGs can affect bone remodeling by regulating macrophage polarization in AS. From the correlation between immune cells, we found a negative correlation between Macrophages M1 and Macrophages M2, which confirms the imbalance of M1/M2 on the other hand (Supplementary Figure S2). Meanwhile, CCL3, PIK3CG, PTGER2 and TNF negatively correlated with neutrophils. Considering that NETs formation was the most significant pathway in AS key module, neutrophils seem to become more remarkable.

## Discussion

In this study, the immune mechanism of LBMD in AS was studied by bioinformatics, community discovery and machine learning. Five immune genes, IFNAR1, PIK3CG, PTGER2, TNF, and CCL3, were defined as the key I-DEGs to illustrate the relationship between AS and LBMD. The signature composed of these had an excellent diagnostic effect on both diseases. We speculate that NETs formation may be the mechanism of AS-induced LBMD. These findings will serve as the foundation for the subsequent discussion.

TNF, also known as TNF-α, is a key regulator of inflammatory response. It has been detected in sacroiliac joints in patients with AS, especially in the early stages of disease activity (Braun et al., 1995; François et al., 2006). Higher serum levels of TNF were associated with the disease activity of AS and played a vital role in the pathogenesis of LBMD in AS (Lange et al., 2000). Moreover, a recent study through the network pathway analysis of differentially abundant proteins indicated disorders of inflammatory signaling pathways in LBMD patients, including the TNF signaling pathway (Al-Ansari et al., 2022). TNF blockers have been widely used in treating AS (Cessak et al., 2014). Denosumab can target RANKL, which is known as TNFSF11 in the TNF superfamily, and treat osteoporosis, which fully illustrates the link between AS and LBMD (Croft et al., 2013). Interestingly, the SNP rs3102734 of the TNF family gene TNFRSF11B was found to be the susceptibility locus of LBMD (Kanehisa and Goto, 2000), genetically confirming the crucial role of TNF, which may be the key to bone loss in AS (Wang et al., 2012).

PIK3CG, also known as PI3Kγ, is a member of the phosphoinositide3-kinase (PI3K) family. It can be activated by G protein-coupled receptors (GPCR) to participate in leukocyte chemotaxis and other immune responses (Barberis and Hirsch, 2008). A recent study demonstrated that PI3KCG stimulates MLCK210, a high molecular weight type of myosin light chain kinase, which activates Rap1 GTP loading and modifies the conformation of integrin α4β1, which promotes tumor inflammation and progression (Schmid et al., 2022). Although the role of PIK3CG in AS has not been confirmed, its inhibitors played an important role in treating autoimmune diseases such as rheumatoid arthritis (Oka et al., 2013), and its role in AS seemed credible. PIK3CG can inhibit the activation of NF-kappaB through Akt and mTOR, thus promoting inflammation (Kaneda et al., 2016). Interestingly, NF-kappaB and its ligand-related RANKL/RANK/OPG system can induce osteoclast differentiation and function in bone resorption (Ono et al., 2020). In addition, it has been confirmed that PIK3CG can act as a mediator of macrophage phagocytosis (Kresinsky et al., 2016) and regulate the release of inflammatory cytokines in macrophages (Lanahan et al., 2022). In our study, PIK3CG was highly expressed and positively correlated with macrophage M1 and negatively correlated with M2. It indicated that the increased expression of PIK3CG in patients with AS led to an imbalance in the proportion of M1/M2, while the bone homeostasis tilted to resorption, coinciding with previous studies. It is possible, therefore, that PIK3CG may be involved in NF-kappaB and regulate the function of macrophages to affect bone homeostasis.

CCL3, as a member of the CC chemokine family, can induce the transport and aggregation of immune cells (Schaller et al., 2017). Under the endothelial-leukocyte interaction, the production of CCL3 was an important mechanism for maintaining cell recruitment during inflammation (Lukacs et al., 1994). Recently, it has been found that the levels of CCl2 and CCl3 in the peripheral blood of patients with rheumatoid arthritis are increased and are positively correlated with rheumatoid factor, which means they have good predictive value for the occurrence, curative effect and prognosis of rheumatoid arthritis (Guo et al., 2021). In the clinical study of AS, CCL3 was widely used to study the disease activity of AS (Akbulut et al., 2010; Pishgahi et al., 2020; Li et al., 2022). CCL3 was the main osteoclast-promoting factor in multiple myeloma, which was mediated by CCR1 (Coniglio, 2018). *In vitro* experiments have shown that high expression of CCL3 in multiple myeloma cells can promote osteoclast maturation in a RANKL-independent manner (Han et al., 2001). It suggests that osteoclast maturation induced by CCL3 may be the mechanism of LBMD in AS.

PTGER2, commonly known as EP2, is the most widely produced prostaglandin in the human body and has multiple effects on various organs, including inflammation, bone healing, and bone formation (Li et al., 2007). Interestingly, AS, a disease characterized by inflammation and new bone formation, is inextricably linked to prostaglandins. A recent study showed that PTGER2 is a crucial potential biomarker of osteogenic differentiation of mesenchymal stem cells, which confirms the osteogenic effect of PTGER2 (Feng et al., 2022). The siRNA of PTGER2 can regulate the expression of sclerosing proteins related to new bone formation in AS (Genetos et al., 2011; Tsui et al., 2014). Genetic polymorphism of the same family gene PTGER4, which was closely related to PTGER2, was associated with AS susceptibility (Chai et al., 2013), and its abnormal expression led to the accumulation of pathogenic Th17 cells and was associated with high disease activity in AS patients (Klasen et al., 2019). PTGER2 also plays a vital role in bone remodeling. It can act as a paracrine factor to affect osteoblasts and osteoclasts, and mediate osteoclast formation with PTGER4 (Jiang et al., 2022). Moreover, PTGER2 can activate PI3K, participate in the Wnt pathway, and prevent osteocyte apoptosis (Kitase et al., 2010).

IFNAR1 (interferon α/β receptor 1) can bind to type 1 IFN, participate in the JAK-STAT transduction cascade, initiate IFN-mediated intracellular signal cascade and regulate immune response (Uzé et al., 2007). IFN pathway is closely related to the pathogenesis of rheumatoid arthritis and induces IFN response genes in synovial fibroblasts after TNF stimulation (Burja et al., 2020). As autoimmune diseases, the pathogenesis of AS and RA is similar, and under this premise, its role in AS seems credible. Ubiquitination of IFNAR1 can limit inflammation-induced tissue damage (Bhattacharya et al., 2014). In addition, the IFN pathway played an inhibitory role in osteoclast formation (Takayanagi et al., 2005). The BMD decreasing and osteoclast production increasing were detected in Ifnar1-deficient mice (Place et al., 2021). In our study, IFNAR1 can participate in osteoclast differentiation, consistently with previous studies, and the role of IFNAR1 in bone balance seems credible.

In addition to the above genes, neutrophil extracellular traps (NETs) formation has attracted our attention as an essential mechanism of AS. On the one hand, in our research, NETs formation was the most significant pathway of the AS key module, and it was the key to the connection between key I-DEGs. On the other hand, our specific immune infiltration in AS showed that neutrophils had the most significant differences between case and control groups and a good correlation with CCL3, PIK3CG, PTGER2, and TNF. NETs are a network of histones, elastases, myeloperoxidase (MPO), and cathepsin G released by neutrophils, which can participate in the process of tumors, infections, autoimmune diseases, and other diseases (Castanheira and Kubes, 2019). NETs can act as an autoantigen to coordinate congenital and adaptive immune disorders, participate in the self-magnifying cycle of autoimmune inflammation, and produce cell and tissue-specific damage in autoimmune diseases such as rheumatoid arthritis and systemic lupus erythematosus (Fousert et al., 2020; Goel and Kaplan, 2020). Similarly, NETs production increased in AS and was associated with AS inflammation and activity (Ruiz-Limon et al., 2018; Zambrano-Zaragoza et al., 2021).

NETs are also closely related to bone remodeling as a vital participant in innate immunity. Carbamylated proteins in NETs can enhance pathogenic immune response, promote osteoclast differentiation and enhance bone resorption (O'Neil et al., 2020). It has been found that Raloxifene, a drug used to prevent and treat postmenopausal women’s osteoporosis, can inhibit NETs production by targeting nuclear estrogen receptors ERα and ERβ (Muchmore, 2000; Hansdóttir, 2008; Flores et al., 2016). Notably, IL-17A-modified NETs can promote MSCs to differentiate into osteoblasts in AS patients (Papagoras et al., 2021). The role of NETs in AS seems to be dialectical. On the one hand, it may promote bone destruction. On the other hand, it may lead to new bone formation in AS. The mechanism is worth further exploring.

In short, immunity seems to be an essential mechanism for connecting AS and LBMD. The key I-DEGs, TNF, CCL3, PIK3CG, PTGER2, and IFNAR1, not only affect neutrophils infiltration, but also participate in neutrophil extracellular traps formation through the pathway such as the MAPK signaling pathway, to involve the bone remodeling process of AS. In addition, the machine learning model composed of key I-DEGs has good diagnostic value for both LBMD and AS, which can guide drug development and clinical management.

### Strengths and limitations

This is the first research to use bioinformatics to evaluate the function of immune-related genes in AS-induced LBMD and investigate the mechanism. Innovative methods such as community discovery and machine learning make the research more exhaustive. We created a comprehensive combination of key I-DEGs and their associated mechanism, filling the gap of the mechanism in previous research and generating novel concepts for future research. Nonetheless, this work was essentially performed by computer technology such as bioinformatics and machine learning, and further experimental validation of this hypothesis is required.

## Conclusion

The key I-DEGs, TNF, CCL3, PIK3CG, PTGER2, and IFNAR1, can be utilized as biomarkers to determine the risk of LBMD in AS patients. They may affect neutrophil infiltration and NETs formation to influence the bone remodeling process in AS.

## Data Availability

The original contributions presented in the study are publicly available. This data can be found here: GSE25101 (https://www.ncbi.nlm.nih.gov/geo/query/acc.cgi?acc=GSE25101); GSE73754 (https://www.ncbi.nlm.nih.gov/geo/query/acc.cgi?acc=GSE73754); GSE56815 (https://www.ncbi.nlm.nih.gov/geo/query/acc.cgi?acc=GSE56815); GSE2208 (https://www.ncbi.nlm.nih.gov/geo/query/acc.cgi?acc=GSE2208). Relevant files and R codes in the study can be directed to the corresponding author.

## Abbreviations

AS, ankylosing spondylitis; AUC, the area under the curve; BP, biological process;DEG, differential expressed gene; GO, Gene Ontology; KEGG, Kyoto Encyclopedia of Genes and Genomes; LBMD, low bone mineral density; LASSO, least absolute shrinkage and selection operator; NETs, neutrophil extracellular traps; PPI, protein-protein interaction; ROC, receiver-operating characteristics; RF, random forest; STRING, Search Tool for the Retrieval of Interacting Genes; SVM, support vector machine; XGBoost, Xtreme Gradient Boosting.
