# Supplementary figures and images for: Immune mechanism of low bone mineral density caused by ankylosing spondylitis based on bioinformatics and machine learning
Source: Front Genet. 2022 Nov 18;13:1054035. doi: 10.3389/fgene.2022.1054035 (PMC9716034; doi:10.3389/fgene.2022.1054035)

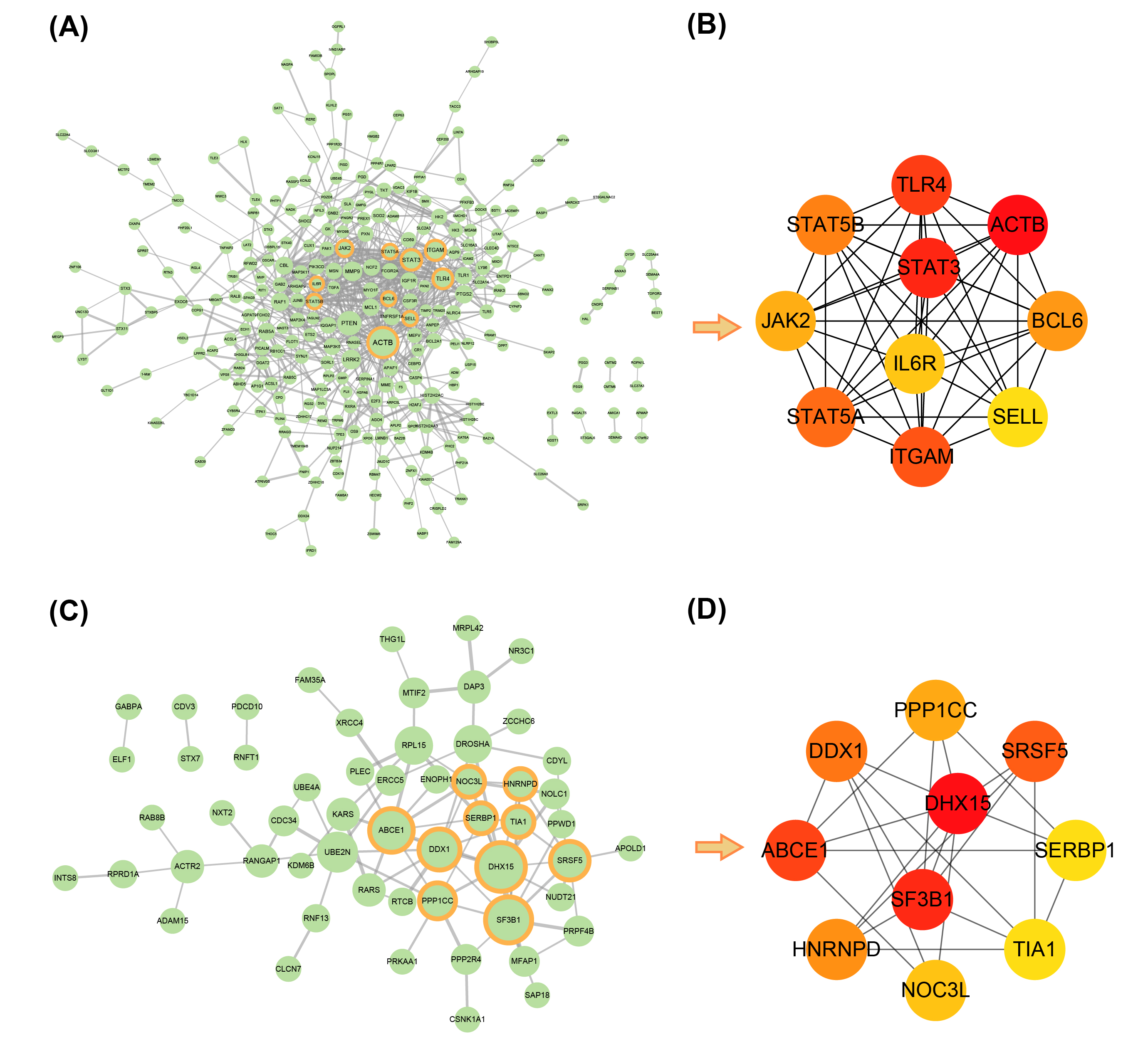

Supplement: Supplementary file 3 [file Image1.JPEG]

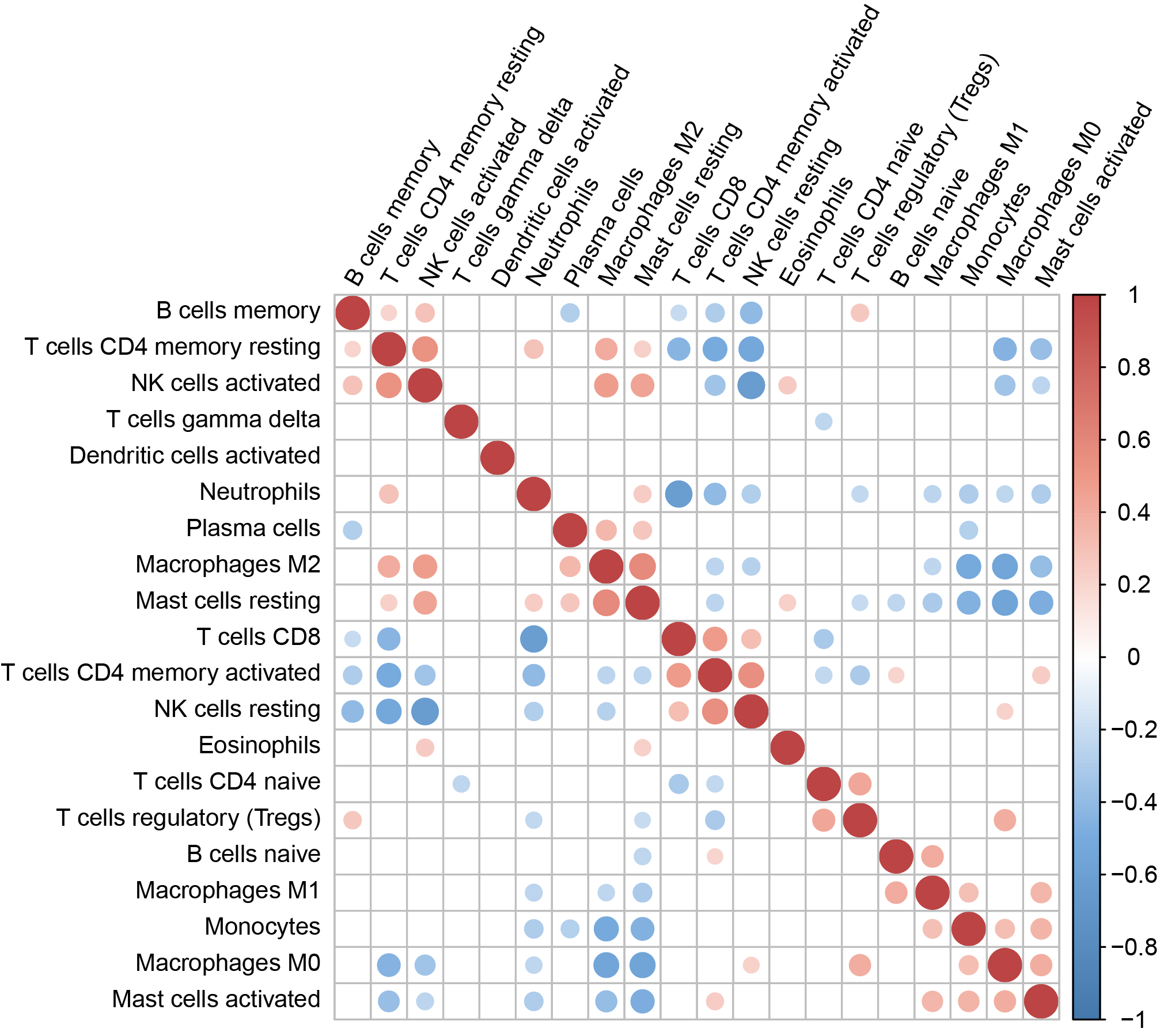

Supplement: Supplementary file 4 [file Image2.JPEG]
